# Bioactive Based Nanocarriers for the Treatment of Viral Infections and SARS-CoV-2

**DOI:** 10.3390/nano12091530

**Published:** 2022-05-01

**Authors:** Ravi Goyal, Rajni Bala, Rakesh K. Sindhu, Mehrukh Zehravi, Reecha Madaan, Sarker Ramproshad, Banani Mondal, Abhijit Dey, Md. Habibur Rahman, Simona Cavalu

**Affiliations:** 1 Department of Pharmacognosy, Chitkara College of Pharmacy, Chitkara University, Rajpura 140401, Punjab, India; ravigoyal7299@gmail.com (R.G.); rajni.bala@chitkara.edu.in (R.B.); reecha.madan@chitkara.edu.in (R.M.); 2 Department of Clinical Pharmacy Girls Section, Prince Sattam Bin Abdul Aziz University, Al-Kharj 11942, Saudi Arabia; mahrukh.zehravi@hotmail.com; 3 Department of Pharmacy, Ranada Prasad Shaha University, Narayanganj 1400, Bangladesh; ramproshad131135@gmail.com (S.R.); banani091110@gmail.com (B.M.); 4 Department of Life Sciences, Presidency University, Kolkata 700073, West Bengal, India; abhijit.dbs@presiuniv.ac.in; 5 Department of Global Medical Science, Wonju College of Medicine, Yonsei University, Wonju 26426, Gangwon-do, Korea; 6 Faculty of Medicine and Pharmacy, University of Oradea, P-ta 1 Decembrie 10, 410087 Oradea, Romania

**Keywords:** anti-viral, bioactive, bioavailability, coronavirus, nano-formulations, phytoconstituents

## Abstract

Since ancient times, plants have been used for their medicinal properties. They provide us with many phytomolecules, which serve a synergistic function for human well-being. Along with anti-microbial, plants also possess anti-viral activities. In Western nations, about 50% of medicines were extracted from plants or their constituents. The spread and pandemic of viral diseases are becoming a major threat to public health and a burden on the financial prosperity of communities worldwide. In recent years, SARS-CoV-2 has made a dramatic lifestyle change. This has promoted scientists not to use synthetic anti-virals, such as protease inhibitors, nucleic acid analogs, and other anti-virals, but to study less toxic anti-viral phytomolecules. An emerging approach includes searching for eco-friendly therapeutic molecules to develop phytopharmaceuticals. This article briefly discusses numerous bioactive molecules that possess anti-viral properties, their mode of action, and possible applications in treating viral diseases, with a special focus on coronavirus and various nano-formulations used as a carrier for the delivery of phytoconstituents for improved bioavailability.

## 1. Introduction

Natural constituents isolated from different plant parts, such as barks, stems, fruits, roots, seeds, and leaves, have been used as herbal medicines for the management of several diseases for over 3000 years. They serve as the leading compound of novel medications in modern drug discovery. More than 40% of anti-virals are natural bioactive molecules or are modified by natural products, such as lead [1]. The World Health Organization (WHO) has reported that approximately 80% of the population uses traditional medicine to treat pathogens [2]. Viral infections, because of their complexity and variety, are one of the major causes of disease and it is impossible to offset its symptoms and its dissemination, which often lead to pandemic events [3]. The pandemic spread of COVID-19 due to extreme acute respiratory syndrome coronaviruses (SARS-CoV-2), which has now passed across borders, is now exacerbating the intensity of viral invasion in more than 190 countries, according to the authoritative data reported by the WHO on 13 April 2022. Countries like Italy (15,467,395 cases), Spain (11,787,004 cases), Germany (23,182,447), and Iran (7,199,861 cases) seem severely affected by a striking invasion of the disease. China has become a global role model in the advocacy of traditional Chinese medicine (TCM), along with conventional COVID-19 therapy, demonstrated by the implementation of multiple randomized studies in patients who reported COVID-19 for validating the safety and effectiveness of TCM. The Indian traditional health system, which has pioneered therapy for infectious diseases for many centuries, has proved its clinical track record in the management of dengue virus through formulations such as Nilavembu Kudineer (NVK), comprising of nine individual herbs. NVK also claims anti-pyretic and anti-inflammatory properties, as per the published records. Another herbal medicine called Kabasura Kudineer (KSK), consisting of 15 herbs, is recommended for managing swine flu by the National Health Portal (NHP), the government of India. Moreover, enlarged migration and urbanization have made the outbreak of viruses a key public health point, particularly due to the lack of vaccines and effective anti-viral therapies [4]. Viruses are marked by a genome (RNA or DNA) enclosed by a protein or lipid envelope that uses cellular reproductive machinery to cause many illnesses, including colds, warts, fever, or even death [5,6]. Due to their genetic variability, special surface molecule configuration, and efficient replication with host resources, they demonstrate different invasion strategies for host cells (Cohen, 2016). Prophylactic technique and/or medical care may be used to combat viral infections. However, viruses remain in the host to reproduce and survive, so most of their metabolic pathways are similar. These aspects highlight the key characteristics of viruses (specificities, affinities, and mechanisms of self-protection), the complexity of anti-viral chemotherapy, and the necessity to explore and classify new anti-virus agents with a selectivity, strength, stability, and toxicity that are primarily relevant [7]. In the immediate aftermath of the Second World War, the production of anti-viral agents began by increasing in vitro and in vivo medicinal plant activity studies, in particular. Moreover, many traditional medicines have failed to combat viral infections and some viral resistances have resulted in a growing interest in plant products as potential anti-viral agents [8]. Several plants and essential oils, as well as isolated bioactive compounds, such as phenolic acids, flavonoids, terpenes, lignans, coumarins, alkaloids, or proteins, showed a potential role as anti-viral agents [9]. So far, many infectious viral diseases have been identified and newer diseases also occur. Among the emerging diseases, most of the diseases include viruses, such as HIV, influenza, herpes simplex virus (HSV), dengue, chikungunya, zika, hepatitis A (HSV), hepatitis B (HSB), hepatitis C (HCV), etc. [10]. Viral diseases are a major health concern since viral infections are difficult to manage due to the viral genomes’ mutative existence [11]. New resistant viral strains are constantly emerging that need new anti-virals with less adverse effects and cell toxicity [12]. In the past, fatal viruses have triggered global pandemics, by increasing the chance of infectious diseases spreading across the continents. Very few medicines have been developed so far to effectively fight against viral diseases [13]. Natural resources are a precious area of science for the study, extraction, and identification of healing properties. A very small proportion of plant chemicals have been explored systematically for their therapeutic potential [14]. It has been found that only 25% of prescription medications currently come from plants [15]. Many anti-neoplastic and anti-infective medicines come from herbal products [16]. There have been about 2500 registered global therapeutic plant species [17,18] to combat many infections and diseases. The bioavailability of natural bioactive molecules in the body is a crucial factor in their bioefficacy, which is restricted due to several reasons, such as rapid metabolism, poor solubility, and permeability due to their large molecular size and poor stability. Various nanoformulations, such as polymeric nanoparticles, carbon nanotubes, lipid nanoparticles, nanoliposomes, nanocrystals, and nano micelles of natural bioactive molecules, are effective in improving the drug biodegradability and biocompatibility and significantly improving effectiveness by removing the pharmacokinetic restriction and site-specific targeting [19]. Nanotechnologies have served a significant impact on nanomedicine throughout the last decade. Nanoparticles have also been utilized in noninvasive imaging techniques to diagnose and monitor illnesses, as well as treatment responses. Nanomaterials can prevent viral binding to the host cell-surface receptor, which is crucial in the context of CoVs. Instead of designing vaccinations, which take a prolonged period and numerous stages of trials, it would be preferable to employ nanotechnology to prevent these viruses from infecting humans. Nanoparticle-mediated delivery methods possess various advantages, such as long-term persistence, lack of enzyme destruction, selective targeting of cells, and upregulation of the immune system. There are significant hurdles in treating infectious illnesses, such as SARS, MERS, and COVID-19, including the lack of effective treatments and vaccinations. Therefore, in 2010, Bachmann and Jennings demonstrated the potency of nanoparticles in enhancing the transport in the lymphatic system in contrast to smaller antigens.

## 2. General Structure of Viruses

**Figure 1:**
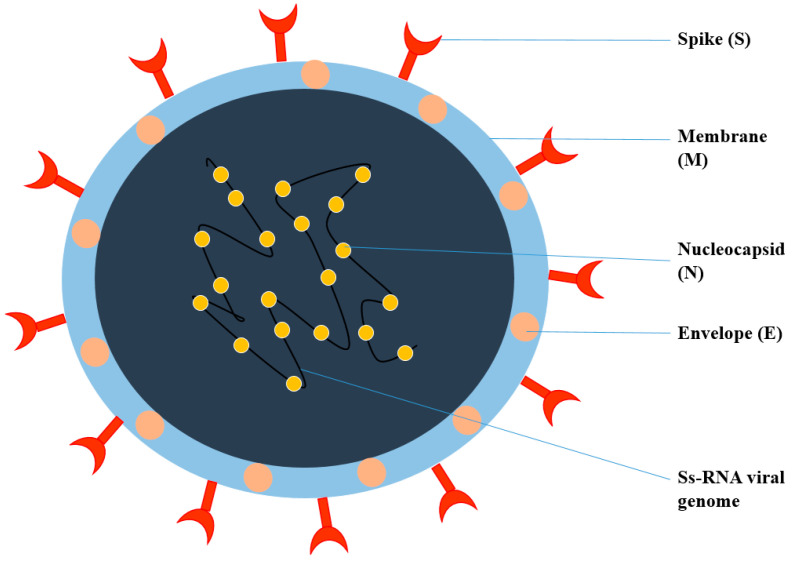
Structure of Coronavirus.

Viruses are intracellular parasites, as depicted in Figure 1, composed of DNA or RNA inside a protein coat that lacks cell walls and cell membranes and does not carry out metabolic processes, so they must attach and enter the host’s cells to use their energy for protein, DNA and RNA synthesis for their survival. It is difficult to kill and manage viral infections, as they live inside the host’s cells and an anti-viral drug that kills viruses may also damage and kill the host’s cells; therefore, for pharmaceutical scientists, it is quite challenging to develop an effective anti-viral drug and vaccine. Therapy for viral diseases using anti-viral drugs is further complicated by the fact that clinical symptoms appear late and until then, most viral particles have been replicated [20]. The key points that need to be considered for the successful development of anti-viral therapy include the development of a molecule that can enter the infected cells to interfere with the nucleic acid synthesis, or to prevent the binding and entry of the virus into the host cells, Most herbal bioactive compounds are known to work efficiently using this strategy, in addition to the synthesis of molecules that strengthen the body’s immune system to combat infection. [21].

Coronavirus is an enveloped, single-stranded RNA virus, known to cause different disorders, such as hepatic, enteric, neurological, and respiratory diseases [22]. Coronaviruses are classified into four genera according to the taxonomic analysis, which includes α, β, γ, and δ coronavirus. The α and β coronavirus assemblies trigger respiratory track information. SARS-CoV-2 falls under β and shares approximately 79.6% of SARS-CoV genome identity [23]. SARS-CoV-2 consists of structural proteins (S, E, M, and N) and non-structural proteins (nsp1−16). The S protein of the virus is a trimeric S glycoprotein that facilitates adhesion to the host cell’s receptor. In most coronaviruses, the S protein is fragmented into two polypeptides called S1 and S2 via the host cell’s furin-like protease. The S1 is a component of the S protein’s large RBD, while the S2 is the stalk of the spike protein structure [23]. The non-structural protein Nsp1 is involved in RNA processing and replication. Nsp2 influences the host cell’s survival signaling system. Nsp3 is thought to be involved in the separation of the translated protein. Nsp4 is a transmembrane domain 2 (TM2) protein that alters ER membranes. In replication, Nsp5 participates in the polyprotein process. Nsp6 is a transmembrane domain. The existence of nsp7 and nsp8 boosted the combination of nsp12 with template-primer RNA, considerably. Nsp9 is a protein that binds to ssRNA. Nsp10 is required for viral mRNA cap methylation. The COVID-19 genome codes four-wide structural and five accessory proteins, including ORF3a, ORF6, ORF-7, OR8, and ORF9, with a size of approximately 29 kb [24]. Coronaviruses replicate their genomes and transcribe their genes via an RNA-dependent RNA polymerase (RdRp) complex. The RdRp complex of SARS-CoV-2 consists of subunits nsp12, nsp7, and nsp8, which enhance their processes and template binding [24].

## 3. Pathophysiology of COVID-19

The three major COVID-19 routes are as follows: (1) transmission by aerosol, (2) transmission by droplets, and (3) transmission by contact [25].

The incidence of COVID-19 infection in individuals is highly impacted by disorders, such as diabetes, hypertension, and lung diseases. This may be attributable to the increased ACE2 receptor expression in several organs, including the kidney, lungs, heart, and host epithelial cells. As COVID-19 enters the human body, it interacts with the ACE2 receptor and releases the RNA inside epithelial cells (EC). It then replicates and is released for further infection and spread via a nasal passage to the alveolar area of the lung [26]. The alveoli are generally the arbitration for gaseous exchange but, because of COVID-19 infection, there is increased permeability, pulmonary edema, disseminated intravascular coagulation (ICD) activation, pulmonary ischemia, hypoxia, respiratory failure, and severe lung damage [27]. It is then transported across the body, including GIT, brain, kidney, liver, and heart, through the blood from the respiratory tract, leading to various neurological disorders, comas, cerebral blood clots, ischemic strokes, and eventually death [28]. COVID-19 infects endothelial cells through ACE-2 binding, causing localized inflammation, endothelial activation, damaging tissue, and impaired release of cytokine. This severe aggregation of cytokine storm through vascular growth factor secretion, monocyte protein-1, interleukin-8, and decreased E-cadherin expression in epithelial cells contribute to vascular permeability and leakages, which are part of the acute respiratory distress syndrome pathophysiology (ARDS). Most Coronavirus-infected patients die from ARDS, in which pulmonary epithelial cells help to begin and transmit ARDS with a shift in the veracity of a vessel’s barrier, encourage the condition of pro coagulation, induce vascular inflammation and reconcile inflammatory cell infiltration [29]. The epithelial cells are the key cause of pathogenesis of ARDS and multi-organ failure in COVID-19 patients, according to the hypothesis. Extreme COVID-19 infection stimulates coagulation mechanisms, which can potentially induce angiogenesis and possible epithelial cell hyperplasia, with the formation of DICs and blockages of small capillaries by the inflammatory cell, as well as significant thrombosis in larger vessels [30,31]. According to Teuwen et al., there are multiple mechanisms proposed for increased vascular permeability and vascular leakage in severely infected patients. The virus may have a direct effect on epithelial cells with widespread epithelial dysfunction, lysis, and death. It binds to ACE-2, decreasing ACE2 activity, indirectly turns on a kallikren–bradykinin route with enhanced vascular permeability to reach the host cells, recruited in pulmonary epithelial cells, stimulated neutrophils to generate cytotoxic mediators, such as reactive oxygen species, immune cells, inflammatory cytokines, and vasoactive molecules contribute to improved contractility and a weakening of inter-endothelial connections in the epithelial cells. The cytokines IL-1β and tumor necrosis factor cause glucuronidases that both degrade glycocalyx and activate hyaluronic acid synthase 2, which results in increased hyaluronic acid deposition within the extracellular matrix and encourages fluid retention [32].

## 4. Natural Anti-Viral Plants and Phytochemicals

Phytoconstituents are naturally isolated bioactive molecules from plant parts, such as vegetables, fruits, flowers, leaves, and roots, that together with nutrients and fibers act as a defense system against disease or, more specifically, help to fight against the disease. Polyphenols, alkaloids, flavonoids, saponins, quinones, terpenes, proanthocyanidins, lignans, tannins, polysaccharides, steroids, organosulfur compounds, and coumarins are protruding bioactive phytochemicals, which have been used in experiments to combat viral infections and have attracted the attention of formulators because of their considerable advantages over synthetic molecules including low toxicity, side effects and low cost and have less potential to develop anti-viral resistance [33]. Phytochemicals are classified into primary and secondary constituents according to their metabolic activities. Primary constituents comprise common sugars, amino acids, proteins, and chlorophyll, while secondary constituents include alkaloids, terpenoids, phenolic compounds, and many more, such as flavonoids, tannins, and so on [34]. Plants have been established naturally over the years in various climatic environments on the planet and are enriched with a wide pharmacokinetic complexity of secondary metabolites. Some of the plants containing natural bioactive constituents are discussed below and play specific roles in combating diverse viral infections and diseases.

### 4.1. Scutellaria baicalensis

*Scutellaria baicalensis* has been used for over 200 years in TCM as a remedy for viral infection and inflammation. The roots of this plant have been used for viral infections, anti-inflammation, and anti-cancer activities. This plant also possesses diuretic, chalagogic and cathartic actions. It contains a variety of sterols, flavonoids, phenylethanoids, essential oils, etc. Its dried roots contain over 30 kinds of flavonoids, including baicalein, baicalin, wagonin, oroxylin A, etc. Baicalin is the most important component and has an anti-SARS coronavirus effect, anti-HIV, free radicle scavenging [35,36,37], etc. Another constituent named oroxylin A has anti-respiratory syncytial viral activity [38]. Current research confirmed that baicalin has in-vitro and in-vivo activity against dengue virus, influenza viruses, and enter virus-71 [39]. The compounds particularly hit cell attachment and intracellular replication of H3N2 and H1N1. In the murine RSV infection model, baicalin significantly reduced macrophages and T-lymphocyte infiltration to the lungs.

### 4.2. Tanacetum vulgare

*Tanacetum vulgare* L. also acknowledged as tansy is an herbaceous plant found in the temperate region of North Africa and Europe. It has been practiced as a traditional medicine that exhibits various properties, such as carminative, anti-diabetic, diuretic, anti-hypertensive, emmenagogue, and so on [40]. The extracts and active compounds isolated from this plant have shown several therapeutic applications, such as anti-bacterial, anti-fungal, anti-oxidant, immunomodulatory, anti-viral, etc. The chemical constituent of this plant includes flavonoids, vacuolar flavonoids (such as apigenin) glycosides, sterols (such as cholesterol and campesterol), triterpenes (taraxasterol and amyrin), and caffeic acid [41]. In 2009, Álvarez demonstrated the anti-viral activity of tansy against the HSV-1 virus from ethyl acetate extract from its aerial parts corresponding to parthenolide isolation [42]. Furthermore, the study revealed the anti-viral properties of methanol extract of the tansy aerial parts and was active against both HSV-1 and HSV-2 viruses [43]. Methanol extract from the blossom of the tansy plant showed anti-tumor properties against potato virus Y (PVY) and cucumber mosaic virus (CMV) [44].

### 4.3. Ruta Angustifolia

*Ruta angustifolia* Pers has been used as a traditional remedy for treating inflammation, curing mal conditions during pregnancy, respiratory problems, musculoskeletal systems, and so on [45]. In Indonesia, it was used prominently for treating liver disease and jaundice. Its chemical constituent consists of angustifolin and aromatic derivatives, including moskachan A, B, C, and D. Other constituents include ostruthin, xanthotoxin, dictamnine, xanthyletin, limonoid, psoralen, etc. [46]. The extract from the leaves of this plant exhibited potent anti-HCV properties. The constituent chalepin and pseudo IX from its leaves inhibited post-entry of HSV, RNA replication and synthesis of viral proteins.

### 4.4. Liriope platyphylla

*Liriope platyphylla* is a perennial plant present across the areas of China, Korea, and Japan and was demonstrated to exhibit biological activities against chronic diseases, such as cough, sputum, and neurodegenerative disorders, obesity, and diabetes [47]. Its chemical constituent spicatoside A interferes with lipopolysaccharide-induced (LPS) activation of extracellular signal-regulated kinases, c-Jun N-terminal kinase (JNK), and NF-kB [48]. It was found that *L. platyphylla* roots contain a principal active moiety that inhibits HBV viral promoter activity via interfering with the NF-kB signaling pathway [49].

### 4.5. Citrus reticulate

*Citrus reticulate* is an evergreen tree with aromatic flowers and glossy leaves that belongs to the family Rutaceae. It is referred to as tangerine, mandarin, or Kamla lebu in Bengal. The existence of different bioactive compounds confirms the uses of this plant for various ailments by traditional practitioners [50]. In 2009, Kirbaslar et al. demonstrated the various chemical components in this plant, which include limonene, myrcene, γ-terpinene, sabinene, and so on [51]. Guo et al. in 2016 and Choi et al. in 2011 demonstrated that, in China, it has been used for treating gastrointestinal and respiratory disorders [52,53]. Tangerine has also been documented to express an inhibitory effect against the respiratory syncytial virus (RSV) [54] and rotavirus replication [55]. Nobiletin, a key bioactive constituent isolated from its pericarps, affects the intracellular replication of RSV. The efficacy of tangerine was also reported against VHF-causing arenavirus entry.

### 4.6. Tinospora cardifolia

*Tinospora cardifolia,* also known as giloya, is an Indian native medicinal plant that belongs to the family Menispermaceace. It is extensively used in Ayurveda because of its therapeutic potential (anti-inflammatory, anti-allergic, anti-diabetic, and immunomodulatory activity). The aqueous extract of *Tinospora cardifolia* boosts immunity in children and is adjuvant to vaccination. In a patient with HIV infection, treatment with *Tinosopra cardifolia* extract is found to reduce the TLC, neutrophile, and eosinophil count, suggesting anti- HIV activity [56]. S. Krupanidhi et al. carried out screening of the phytochemicals present in Tinospora cardifolia for an inhibitory effect against SARS-Cov-2. Based on the study, the phytochemicals berberine, tinosponone, xanosporic acid, and tembetarine were identified as the possible lead compounds that can be considered to have activity against SARS-Cov-2. The research proved tinosponone as a potent, selective, and non-toxic inhibitor of 3 CL protease of SARS-Cov-2 [57].

### 4.7. Andrographis paniculata

*Andrographis paniculata,* generally known by the name of kalmegh, is one of the most commonly used medications in Ayurveda, belonging to the family Acanthaceae. The principal constituents present in this plant are flavonoids, diterpenoids, and polyphenols. The major bioactive compound responsible for its therapeutic activity is andrographolide [58]. Both 25µg/mL of ethanolic extract of *Andrographis paniculata* and 5 µg/mL of Andrographolide demonstrated anti-viral activity against a variety of viruses, including influenza A virus, hepatitis B and C, herpes simplex virus, Epstein–Barr virus, and human immunodeficiency virus. They inhibit viral entry inside the viral cell and prevent the replication of genetic material by DNA and RNA polymerase, protein synthesis, and functional mature proteins [59]. Du et al. used alcohol as a co-surfactant, tween 80 as a surfactant, isopropyl alcohol as an oil phase, and water to prepare an andrographolide-loaded microemulsion [60]. Sermkaew et al. used caproyl, cremphor, and labrasol [61], while Syukri et al. used caproyl, tween 20, and polyethylene glycol 400 for the formulation of microemulsions. It was concluded in all the studies that the solubility and bioavailability of andrographolide were increased significantly [62].

### 4.8. Saururus chinensis

*Saururus chinensis*, belonging to the family Saururaceae, is a perennial herbaceous plant found in Korea and China. Traditionally, it has been used as an anti-pyretic, anti-inflammatory, and diuretic agent. A phytochemical investigation led to the discovery of various compounds, including flavonoids, aristolactamus, furanoditerpens, anthraquinones, etc. [63]. Lignans are the main bioactive constituent of plants that exhibit various activities, such as HIV-1 protease, NF-kB, and HIF1 inhibitory effects. Manassantin B, a bioactive compound extracted from the roots of Saururus, reported inhibitory activity against Epstein–Barr virus (EBV) lytic replication [64]. Some of the plants with anti-viral activities, along with their phytochemicals, have been summarized in Table 1.

## 5. Mechanism of Action of Bioactive Molecules in Various Viral Diseases

**Figure 2:**
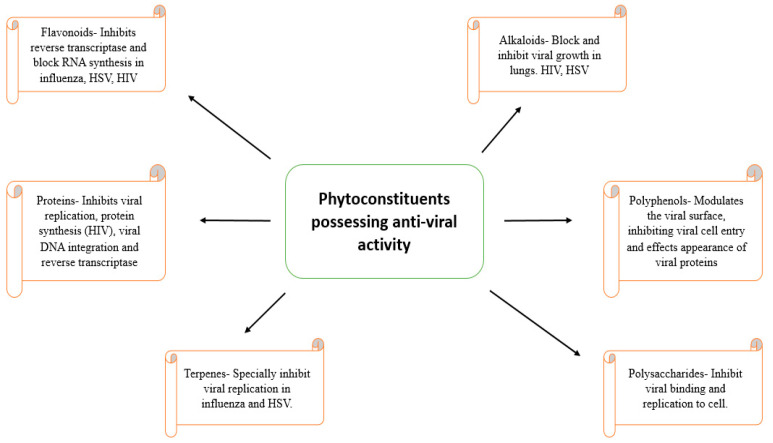
The activity of anti-viral phytomolecules with their mechanisms of action.

The development of resistance against the available synthetic anti-viral molecules necessitates the need to search for more effective compounds against viral infection. Traditional medicinal plants have been known for their potential to fight against viral infections [71,72]. Medicinal plants are reported to have a variety of chemical constituents, which have the potential to inhibit the replication cycle of DNA and RNA [73]. Some of these include alkaloids, which inhibit viral growth, lectins, which inhibit virus penetration, and flavonoids, which hinder reverse transcriptase and viral protein synthesis in HIV [74]. Cadman in 1960 demonstrated that polyphenol extracts from the leaves of *Rubus idaeus* act by clumping the virus particles together into complexes, making it non-effective. In addition, in 1986, Berghe et al. proposed that polyphenols exert their effect via binding to a virus and can stop its absorption into the cell membrane. Furthermore, Sakagami et al. in 1995 suggested that polyphenols mainly act via inactivating the virus and viral replicating enzymes. Bioflavonoid extract, such as baicalein, possesses anti-viral activity via interfering with the interaction of HIV-1 envelopes with chemokine co-receptors and blocking its HIV-1 entry of the target CD4 cells. The mechanism of the binding of flavonoids involves the inhibition of the synthesis of viral DNA. Potent anti-HIV flavonoids, such as quercetin, baicalein, and myricetin, showed inhibitory activity against cellular DNA or RNA polymerase [75]. Many lignans that show anti-viral activity have been identified, such as lignans isolated from *Rhinacanthus nasutus* and *Kadsura matsudai* expressed anti-hepatitis and anti-HIV activity [76]. Moronic acid, a triterpenoid extracted from *Rhus favanica*, exhibited oral therapeutic efficacy against HSV-infected mice [77]. Triterpene acids, such as masalinic and ursolic acid, expressed a potent inhibitory effect against HIV-1 protease [78]. Various anthraquinone extracts of *Aloe barbadensis, Rhamnus purshianus*, *Cassia angustifolia,* etc. showed potent activity against HSV-1 [79]. In 1996, Marchetti et al., revealed that scleroglucan polysaccharide binds with membrane glycoproteins of viral particles and impedes the complexation of the virus with the plasma cell membrane [80]. The activity of anti-viral phytomolecules with their mechanisms of action has been summarized in Figure 2.

## 6. Role of Essential Oils in Combating Viral Infection

**Figure 3:**
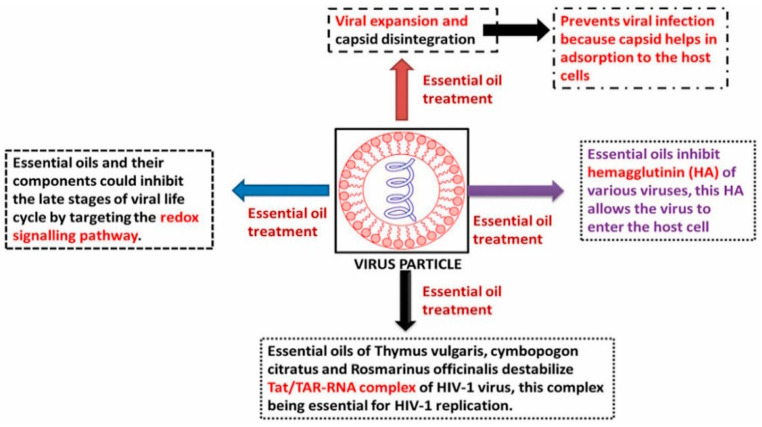
Proposed mechanism of action of various essential oils for anti-viral activity.

Essential oils are non-polar compounds with anti-microbial properties found in number of herbs, including spices. These are generally used in food to impart flavor as they contain volatile compounds monoterpenes (such as limonene, vanillin, eugenol, and safrole), and monoterpenoids (thymol, borneol, carvacrol, and citronellal). The anti-viral action of many essential oils has been studied and shows that they possess anti-viral activity against both enveloped and nonenveloped viruses, including HSV, influenza, adenovirus type III, and poliovirus [81]. Oils from eucalyptus, melaleuca alternifolia, eugenol, thymol, and camphor exhibited anti-viral activity against HSV-1 and HSV-2 [82]. Sandalwood oil, extracted from Santalum albumin, exhibited dose-related activity against HSV-1 [83]. Black seed oil from Nigella sativa showed a striking effect against MCMV infection. The proposed mechanism by which various essential oils act is given in Figure 3.

## 7. Herbal Strategies to Combat COVID-19

**Figure 4:**
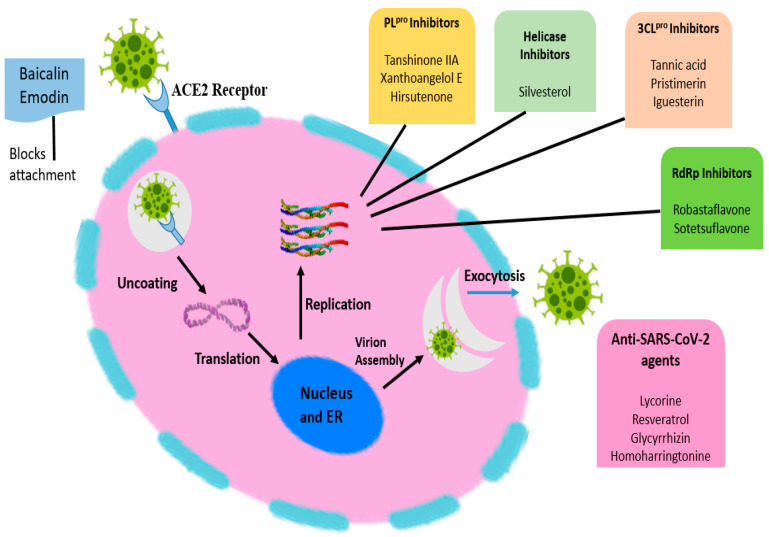
Role of enzyme and protein inhibitors in SARS-CoV.

**Figure 5:**
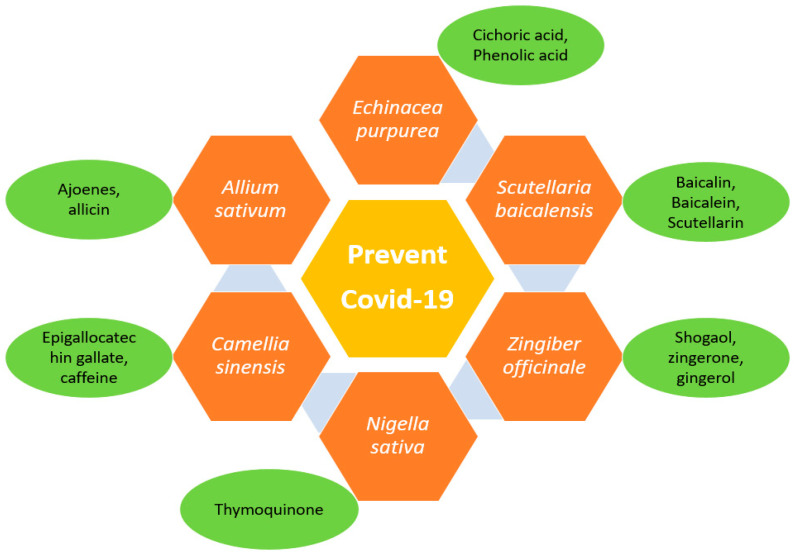
Herbs are effective in preventing COVID-19 infection.

Natural products and their phytoconstituents isolated from them selectively block the ACE2 receptor without blocking enzyme activity, which may be useful in the treating and spreading of viral infection without increasing ACE2 expression in patients and, therefore, increasing the risk of viral infection. The higher ACE inhibitory potential of various natural medicinal species might be due to their high phenolic content and higher antioxidant potential. The role of enzyme and protein inhibitors in SARS-CoV is shown in Figure 4. Different traditional herbs from different geographical locations, as depicted in Figure 5 and are used in the treatment of SARS-COV2 also. Various herbal and fruit extracts were claimed to be a remedy for viral infections. Studies reported that the usage of Andrographis paniculata lowers the symptoms of coronavirus and acts as an immune booster [84]. Furthermore, the drinking of warm limewater has been reported to prevent COVID-19 disease, by raising the levels of vitamin C in the body. However, according to WHO, there are no such claims but suggested the consumption of fresh fruits [85]. According to research, *Aloe vera* (L.) Burm. f. gel and its constituents, such as aloin and aloe-emodin, inhibit enveloped viruses including SARS-CoV-1, HIV, and influenza via blocking viral replication or destroying the lipid envelope. Studies showed that phytoconstituent concanavalin A and a phytoagglutinin in jack beans could attach to glycosylated membrane proteins and prevent viral entry [86]. Several medical extracts, including *Artemisia annua*, *Lindera aggregate*, *Lycoris radiata*, etc. exhibited an anti-SARS-CoV effect [87]. Lau et al. showed that aqueous Houttuynia cordata can hinder the efficiency of two proteins, namely the chymotrypsin-like protease and RdRp, in SARS-CoV [88]. Various active constituents of medical plant extracts, such as *Polygonum multiflorum, Rheum officinale*, etc., inhibited the binding of the SARS-CoV (S) spike protein to ACE 2 [89]. Additionally, some active phytoconstituents, such as two triterpenes, two sesquiterpenes, curcumin, and five lignans suppressed SARS-CoV. Emodin extract from Rheum and Polygonum, nicotinamide from foodstuff, baicalin from *Scutellaria baicalensis*, scutellarian and luteolin from *Veronicalina riifolia* inhibited a SARS- CoV S-protein and ACE 2 interaction [90]. Baicalin typically reduces the oxidation damage in cells induced by angiotensin II and activates the ACE2-Ang (1-7)-Mas pathway [91].

After the outburst of COVID-19, CTM played a significant role in controlling COVID-19 [92]. Due to the low toxicity and widespread availability of CTM, the isolation of bioactive compounds against viral or host targets from CTM might be a promising technique for treating COVID-19. As per the Guideline for the Diagnosis and Treatment of Novel Coronavirus Pneumonia (On Trials, the seventh edition) in China, *Qingfei Paidu* decoction is approved for the treatment of clinically verified cases. A study of 98 patients with COVID-19 found that *Qingfei Paidu* decoction has a favorable therapeutic impact on COVID-19 therapy and minimized the adverse effects. Furthermore, Lianhua Qingwen was shown to drastically inhibit SARS-COV-2 replication at the mRNA level in Vero E6 cells and exhibited a potent inhibitory effect on the cytokine storm caused by SARS-COV-2 [93]. Research has demonstrated that some of the herbal formulas could be potent in disease treatment. Sini decoction, consisting of ginger rhizome, licorice, and aconite, could be effective in dealing with COVID-19, as it prominently ameliorated *E-coli*-induced lung injury by reducing the inflammatory factors in lung tissue and inhibiting the expression of Ang II type 1 receptor and ACE [94,95]. Silvestrol, which is isolated from the plant belonging to the genus Aglasia, can be introduced as a good remedy for COVID-19 treatment, since it showed potent anti-viral activity in Ebola virus-infected human macrophages, suppressing elF4A-dependent viral mRNA translation [96,97]. Resveratrol, which is a stilbenoid, showed an inhibitory effect against MERS-CoV in vitro. Therefore, it can be used as a capable remedy against COVID-19 [98,99]. Another flavonoid named luteolin is a prominent anti-viral drug against COVID-19, as it interfered with viral entry into the host through binding to the spike protein of SARS-CoV [100,101]. According to a study, a leaf extract of *Toona sinensis* from the plant *Cedrela sinensis* exhibited an evident effect against SARS-CoV. Therefore, this vegetable can be explored as a new anti-viral drug against SARS-CoV-2 [102,103]. *Withania somnifera* (L.) and its compounds, such as Withaferin-A and Withaone, prevent SARS-CoV-2 invasion into host cells via binding to TPMRSS2 [104].

As a consequence of SARS-CoV-2 infection in the lungs, the overexpression of RAC/CDC42 (P21)-activated kinase 1 (PAK1) is a major modulator of the cytokine storm, which often raises mortality in patients. Propolis-derived substances reduce pro-inflammatory NK cellular proliferation, PAK1 activation, cytokine surplus, enhance NF-kB and increase the antibody response against SARS-CoV-2 [105]. According to studies, xanthorrhizol, a phytoconstituent from the Java turmeric plant, might be utilized to treat COVID-19, due to its potential to block pro-inflammatory cytokines [106]. EGYVIR, a combination of curcumin and black pepper extract, showed a promising anti-viral effect against SARS-CoV-2 via the downregulation of the nuclear translocation of NF-kβ p50 [107].

## 8. Anti-Viral Bioactive-Based Nanocarrier Systems

Natural medicines can give beneficial and promising therapeutic results when incorporated into pharmaceutical nanotechnology. Boosted clinical and therapeutic responses are possible with nanoformulations used as carriers for delivering poorly soluble phytoconstituents and plant extracts [108]. Various delivery systems, such as self-nano emulsifying drug delivery systems (SNEDDS), hydrogels, phytosomes, microspheres, transferases, etc., have been used for the delivery of phytoconstituents with anti-viral potential. These nanoformulations displayed numerous effects, such as improved oral solubility, systemic bioavailability, delayed metabolism, and enhanced therapeutic activity. For example, chitosan nanoparticles containing catechin and EGCG resulted in an enhanced rate of intestinal absorption [109]. According to studies, myricetin, a natural flavonoid, was loaded into a polymeric nano-particle carrier, which significantly increased its solubility profile [110]. The encapsulation of flavonoids into red blood cells can boost anti-viral activity and bioavailability. The flavonoids showed positive impacts in reducing oxidative damage to the erythrocyte membrane [111]. Several researchers reported that RBCs play a vital role in the distribution and bioavailability of circulating quercetin [112]. Surprisingly, flavonoids in chitosan particles retain their anti-oxidant activity and can be used to combat free radicals in the bloodstream [113]. The most evenly distributed type of polylactic acid-4 nanoparticle was effectively used to encapsulate quercetin, which permitted the delayed release of quercetin [114]. Kim et al. [115] boosted the oral bioavailability of apigenin by incorporating apigenin into the water-in-oil emulsion system. Due to the poor solubility and permeability of baicalein, Zhang et al. [116] used a micellar composition including the carriers Pluronic P123 copolymer and sodium taurocholate, which significantly increased oral absorption of baicalein. Oleanolic acid was incorporated into SMEEDS, which enhanced its systemic bioavailability [117]. Andrographolide has poor absorption properties and limited oral bioavailability. To circumvent these constraints, PLGA was used to formulate andrographolide-loaded microspheres [118]. According to studies, methanolic extracts of strawberries (*Fragaria ananassa* Duch.) and ginger (Zingiber officinale) were utilized to synthesize silver nanoparticles (AgNPs) to investigate their SARS-CoV-2 inhibitory capability [119]. Several studies are being carried out on the development of nanotechnology for Indonesian Jamun to fight against SARS-CoV-2 [120]. Various other formulations containing anti-viral phytoconstituents are summarized in Table 2.

## 9. Future Prospective and Conclusions

Viral infections remain a major worldwide cause of morbidity and mortality. Among the most aggressive viral infections are Ebola, acquired immunodeficiency syndrome, influenza, and SARS (severe acute respiratory syndrome). For instance, influenza is responsible for over 3 million new cases of severe disease, and between 300,000 and 500,000 deaths yearly [128]. It is difficult to treat various viral diseases and infections without effective vaccines and specific anti-viral therapy. Nonetheless, bioactive compounds serve as an excellent source of biodiversity for discovering efficacious novel antivirals drugs, revealing new structure-activity relationships, and developing effective protective/therapeutic strategies to fight the battle against viral infections. Numerous natural products, along with essential oils and herbal constituents isolated from them, are observed to possess the strong potential to fight against viral infections and their discoveries can provide further help in synthesizing derivatives and therapeutic leads. As large research studies in this area are only preliminary, further details of experimentation in characterizing the bioactive constituent, defining the principal mechanisms, as well as evaluating the efficacy and in vivo studies, are encouraged to develop more therapeutically sound anti-viral therapies through natural products. Furthermore, additional studies need to be performed to explore the possibility of developing combination therapies with other natural agents, such as polyherbal nanoformulations with site-specificity, which may help in reducing the risk of developing drug-resistant viruses. Phytopharmaceuticals will continue to play an important role and contribute to novel nanoformulations as a carrier for safe and cost-effective delivery systems.

## Figures and Tables

**Table 1:** Plants with anti-viral activities, along with their phytochemicals.

Plant	Part	Phytochemical	Class	Active against Virus	Reference
*Ziziphus jujuba*	Roots	Jubanines	Alkaloids	PEDV	[65]
*Rheum palmatum*	Roots	Sennoside A	Glycoside	HIV-1	[66]
*Swietenia macrophylla*	Stem	Limonoids	Lignin	HSV	[67]
*Embelia ribes*	Seeds	Quercetin	Flavonoid	HSV	[68]
*Humulus lupulus*	Whole plant	Xanthohumol	Chalcone	BVDV	[69]
*Glycyrrhiza inflate*	Roots	Chalcones	Ketone	Influenza A	[70]

**Table 2:** Various other formulations contain anti-viral phytoconstituents.

Active Phytoconstituent	Formulation	Applications	References
Oxymatrine	Phytosome	Enhanced bioavailability	[121]
*Artemisia arborescens*	Liposomal	Raised anti-viral activity and improved stability	[122]
Hypocrellins	Nanoparticulate	Enhanced hydrophilicity and stability	[123]
Matrine	Emulsion	Enhanced sustained released activity	[124]
Quercetin	Microsphere	Enhanced bioavailability and sustained release formulation	[125]
Curcumin	Nanostructured solid lipid carrier systems	Improved mucoadhesion and mucus penetration	[126]
Honokiol	Cyclodextrin inclusion complexes	Enhanced solubility and bioavailability	[127]

## Data Availability

The data supporting the findings of this study are available within the article.

## Abbreviations

SARS-CoV-2	Severe acute respiratory syndrome coronavirus 2
WHO	World Health Organization
TCM	Traditional Chinese medicine
NHP	National Health Portal
HSV	Herpes simplex virus
SARS	Severe acute respiratory syndrome
MERS	Middle East respiratory yndrome
TM2	Transmembrane domain 2
RdRp	RNA-dependent RNA polymerase
ORF	Open reading frame
ACE2	Angiotensin-converting enzyme 2
ICD	Disseminated intravascular coagulation
ARDS	Acute respiratory distress syndrome
IL	Interleukin
H3N2	Influenza A virus
PVY	Potato virus Y
CMV	Cucumber mosaic virus
HCV	Hepatitis C virus
LPS	Lipopolysaccharide
JNK	c-Jun N-terminal kinase
NF-kB	Nuclear factor kappa B
RSV	Respiratory syncytial virus
EBV	Epstein–Barr virus
HIV	Human immunodeficiency virus
TPMRSS2	Transmembrane serine protease 2
PAK1	P21 (RAC1) activated kinase 1
SNEDDS	Self-nanoemulsifying drug delivery systems
EGCG	Epigallocatechin gallate
RBC	Red blood cell
PLCA	Poly lactide glycolic acid
